# Comparing Invasive Pulmonary Aspergillosis Mortality Between Liposomal Amphotericin B and Voriconazole in Patients With Hematological Malignancy or Hematopoietic Stem Cell Transplantation

**DOI:** 10.7759/cureus.31762

**Published:** 2022-11-21

**Authors:** Ebrahim Mahmoud, Mohsen Alzahrani, Shukri Loutfi, Hajar Y Alqahatani, Mohammad Bosaeed, Ayoub Ahmed, Bader Alahmari, Husam Alsadi, Mazin Ahmed, Mohammed Al Dhoayan

**Affiliations:** 1 Medicine, King Abdulaziz Medical City Riyadh, Riyadh, SAU; 2 Medicine, Ministry of National Guard-Health Affairs, Riyadh, SAU; 3 Oncology, King Abdulaziz Medical City Riyadh, Riyadh, SAU; 4 Radiology, King Abdulaziz Medical City Riyadh, Riyadh, SAU; 5 Pharmacy, King Abdulaziz Medical City Riyadh, Riyadh, SAU; 6 Hematology, King Abdulaziz Medical City Riyadh, Riyadh, SAU; 7 Health Informatics, King Saud Bin Abdulaziz University for Health Sciences, Riyadh, SAU

**Keywords:** outcome, hematological malignancy, liposomal amphotericin b, voriconazole, invasive pulmonary aspergillosis

## Abstract

Objectives

We evaluated liposomal amphotericin B versus voriconazole for the treatment of invasive pulmonary aspergillosis (IPA) in patients with hematological malignancy or hematopoietic stem cell transplantation (HSCT).

Methods

This retrospective cohort, single-center study included patients with compatible radiological diagnosis of IPA between 2016 and 2021.

Results

Forty-six patients with hematological malignancy or HSCT were diagnosed with IPA. Thirty-nine of them fulfilled the criteria for comparing liposomal amphotericin B (n=15) with voriconazole (n=24). Their median age was 48.5 years. Stem cell transplant recipients were 45.65%, and nearly half of the patients (47.83%) had acute myeloid leukemia. Twenty-six (56.52%) of the patients did not require oxygen therapy. The 12-week mortality was 13.33% (two out of 15) in patients who received liposomal amphotericin B compared to 25% (six out of 24) in patients who received voriconazole. There was no mortality judged to be related to IPA. Success or global clinical response was not different between the two drugs: 80% for liposomal amphotericin B versus 83.33% for voriconazole. However, the safety profile favored liposomal amphotericin B.

Conclusion

In this small cohort, there was an equipoise in the mortality and clinical and radiological outcomes obtained using liposomal amphotericin B or voriconazole for the treatment of IPA in hematological malignancy or HSCT.

## Introduction

Invasive pulmonary aspergillosis (IPA) is an opportunistic fungal infection affecting the immunocompromised population primarily. It is a common complication in patients with hematologic malignancies and/or hematopoietic stem cell transplantation (HSCT) [1]. Its incidence ranges from five percent to more than 20 percent in high-risk groups [2,3]. IPA is associated with elevated hospital mortality, extended duration of hospitalization, and high costs [4]. 

Concerning therapy, voriconazole is currently recommended for the treatment of IPA on the basis of the results of a randomized trial in which it was associated with improved outcomes compared with amphotericin B deoxycholate [5]. The option of voriconazole has been recommended as a first line in the treatment guidelines [6].

While voriconazole is an azole drug that blocks the synthesis of ergosterol [7], amphotericin B is a polyene macrocyclic antifungal molecule generated by Streptomyces nodosus [8]. Lipid-supplemented derivatives of amphotericin B have been introduced to reduce the renal toxicity of the drug and enable more extensive administration [8,9].

There is a paucity of real-life data for IPA in hematological malignancy outside the clinical trial setting worldwide, as we are lacking real-life data regarding the outcome of IPA using different therapies, including liposomal amphotericin B [10,11]. This is also true regarding the evidence of IPA from the region, including epidemiology data. Most of the evidence is related to invasive candidemia rather than aspergillosis, with no specific study related to IPA [12,13].

Thus, we conducted this study to explore the difference in the outcome of IPA using voriconazole versus liposomal amphotericin B.

## Materials and methods

Study design and setting

This is a retrospective cohort study conducted at King Abdulaziz Medical City (KAMC), which is a tertiary care center in Riyadh, Saudi Arabia, with over 1500 bed-capacity, providing care to a diverse patient population. The hematological division provides care for a large number of patients, including those with hematological malignancies and HSCT recipients. Ever since the HSCT program started in 2010 at our center, more than 700 adult patients have undergone transplants.

Study population and definition

Adult patients with hematological malignancy or HSCT recipients diagnosed with IPA, based on radiological criteria in chest computed tomography (CT), who had received either liposomal amphotericin B or voriconazole between January 2016 and September 2021 were included in this study.

Data collected from the patient records included demographics data, clinical presentation data, diagnosis data, investigations requested, the severity of disease, mortality, therapy, as well as microbiologic data, including cultures and galactomannan test results.

A consultant radiologist, Shukri Loutfi (SL), specializing in lung imaging, reviewed all the CT chest scans, which was done initially to ensure compatibility with the diagnostic criteria of IPA [14]. The radiological criteria based on CT chest required one of the following to be present: dense, well-circumscribed lesions(s) with or without a halo sign, air-crescent sign, or cavity. Additionally, SL assessed the follow-up CT if performed between two and six weeks after the diagnosis.

The clinical response assessment follows the defining of the responses to therapy by the 2008 European Organization for Research and Treatment of Cancer/Mycosis Study Group (EORTC/MSG) criteria [15]. Complete response was defined as resolution of all signs and symptoms and more than 90% radiographic improvement compared with baseline; the partial response was defined as clinical improvement and more than 50% radiographic improvement compared with baseline; stable disease was defined as no change from baseline, or less than 50% radiologic improvement; failure was defined as progression of the disease, or not meeting any of the aforementioned categories [15]. Global response or success included complete or partial responses.

The inclusion criteria in our study were adult patients (older than 14 years) with hematological malignancy or stem cell transplant recipients with confirmed radiological diagnosis of IPA. Being on a mold-active agent (posaconazole or voriconazole) as prophylaxis was allowed. Patients were excluded if there was a previous IPA diagnosis and treatment, or the presence of other fungal infections such as zygomycetes infections. For comparing liposomal amphotericin B with voriconazole, the following patients were excluded: those who did not complete seven days of antifungal treatment were receiving conventional amphotericin, or had received dual antifungal treatment for more than 96 hours. Patients who were switched from one medication to another (voriconazole or liposomal amphotericin B) were included if they had been on a single antifungal treatment for fewer than seven days.

The Platelia™ Aspergillus Ag (Bio-Rad Laboratories Inc., Hercules, USA), an immunoenzymatic sandwich microplate assay, was used for the detection of Aspergillus galactomannan antigen in serum and bronchoalveolar lavage (BAL) fluid, and it reported the qualitative result as positive or negative.

Outcome

The primary outcome of this study was to evaluate the 12-week mortality difference between liposomal amphotericin B and voriconazole for 39 patients. Secondary objectives included the evaluation of six-week mortalities and the success/global response difference between the two drugs. Additionally, among the secondary objectives of the study were the evaluation of the clinical presentation, severity, and risk factors of mortality at 12 weeks based on positive galactomannan, neutropenia, and HSCT recipient status.

Statistical analysis

We reported categorical data as frequencies and percentages, while continuous data were reported as mean ± standard deviation. Univariate logistic regression models were used to investigate the significance of the difference between the two medications on multiple factors. All the factors found to be significantly different at a level of p-value 0.05 were included in a multiple logistic regression model with a level of significance at 0.05. A similar analysis was conducted to investigate the significance of the association between these factors and the 12-week mortality.

Ethics and consent

The study was approved by the Institutional Review Board (IRB) at King Abdullah International Medical Research Center (KAIMRC; protocol number: RC 19/264/R). Written informed consent was waived by the IRB, as the study was a retrospective chart review where research involves no more than minimal risk to the subjects. The study complied with the Declaration of Helsinki concerning maintaining the confidentiality of the patient's data as the data were anonymized.

## Results

Between January 2016 and September 2021, 46 patients with hematological malignancy or HSCT were diagnosed with IPA. However, only 39 patients were included in the comparison analysis between liposomal amphotericin B (n=15) and voriconazole (n=24).

Their median age was 48.5 years, and 25 (54.35%) of patients were males. The majority (80.4%) of radiological finding was dense, well-circumscribed lesions(s) with or without a halo sign in 37 patients, followed by the presence of a cavity in seven (15.2%) and an air-crescent sign in two patients (4.1%).

Regarding the patients' comorbidities, the most common hematological etiology was acute myeloid leukemia (AML) in nearly half of the patients (47.83%), and 34.78% had lymphoma. Out of all patients, 45.65% were neutropenic (absolute neutrophil count <0.500×109 cells/L) at the time of IPA diagnosis. The HSCT recipients comprised 45.65% of the total population. Table 1 presents more characteristics of the patients.

**Table 1:** Baseline demographic characteristics, underlying diseases and IPA *Voriconazole - 24 patients, liposomal amphotericin B - 15 patients, and seven others (four patients received dual antifungals, two patients received antifungal treatment for more than six days before the diagnosis, one died within four days of diagnosis) **All the included patients are considered possible IPA IPA - invasive pulmonary aspergillosis

Patients characteristics	n=46 (%)*
Age, years (median)	48.5
Gender, male	25 (54.35%)
Neutropenia, absolute neutrophil count <0.500×109 cells/L	21 (45.65%)
Comorbidities	
Liver disease	1 (2.17%)
Congestive heart failure	1 (2.17%)
Renal disease, estimated glomerular filtration rate (eGFR) <60 ml/min	1 (2.17%)
Stroke/neurological disease	4 (8.70%)
Diabetes mellitus	7 (15.22%)
Underlying diseases/conditions	
Hematopoietic stem cell transplant	21 (45.65%)
Acute myelogenous leukemia	22 (47.83%)
Lymphoma	6 (13.04%)
Acute lymphoblastic leukemia	7 (15.22%)
Chronic myeloid leukemia	2 (4.35%)
Chronic lymphocytic leukemia	2 (4.35%)
Myelodysplastic syndrome	2 (4.35%)
Multiple myeloma	2 (4.35%)
Other	3 (6.52%)
Diagnosis of IPA**	
Proven (based on pathology)	1 (2.17%)
Probable: galactomannan antigen in serum or bronchoalveolar lavage fluid	9 (19.5%)
Positive respiratory culture	(4.35%)
Trigger for the diagnosis	
Fever only	16 (34.78%)
Respiratory symptoms only	8 (17.39%)
Fever and respiratory complain	17 (36.95%)
Other	5 (10.87%)
Severity of disease	
Mild (not required oxygen therapy)	25 (54.35%)
Moderate (required oxygen therapy)	15 (32.61%)
Severe (required ventilation; non-invasive or mechanical ventilation)	6 (13.04%)
Use of systemic antifungal agents	
Antifungal prophylaxis	18 (39.13%)

Clinical characteristics

The main trigger for the diagnosis of IPA was fever and respiratory complaints in 36.95% of the patients, followed by fever alone in 34.78%. A total of 18 (39.13%) patients were receiving antifungal prophylaxis; 11 patients were on fluconazole, seven patients were on the mold-active agent, three patients were on posaconazole, and four were on voriconazole.

The severity of the illness within six weeks of diagnosis was as follows: more than half of the patients (54.35%) had mild disease and did not require oxygen supplement, and six patients (13.04%) required ICU transfer: non-invasive ventilation or mechanical ventilation.

Diagnosis

In our case, a compatible radiological diagnosis of IPA (by the radiologist) was required for case definition, in addition to a risk factor of hematological disease or HSCT. Thus, all the cases fulfilled the criteria of possible IPA as per the guidelines.

The following were the cases of probable IPA: positive galactomannan was found in nine patients (19.5%). Seven patients (15.2%) had a positive test in serum, and four (8.6%) patients had a positive test in BAL; two patients had tested positive for both. Only one patient had a proven diagnosis of IPA as per lung biopsy findings, while two patients grew Aspergillus from respiratory culture.

Antifungal treatment and outcome

Fifteen patients received liposomal amphotericin B, and 24 patients received voriconazole. Seven patients were excluded from the comparison analysis because one patient died within four days of diagnosis, four patients received dual antifungal for more than 72 hours, and two patients received antifungal for more than six days before the diagnosis.

The median duration of antifungal therapy was 76.5 days, including some of the patients who continued on antifungals as secondary prophylaxis. Out of all patients, 69.6% were on antibiotics at the time of diagnosis, and one-third of the patients (32.6%) were on the carbapenem class of antibiotics (meropenem or imipenem).

Comparing voriconazole versus liposomal amphotericin B

Comparison of voriconazole and liposomal amphotericin B are presented in Table 2.

**Table 2:** Comparing outcomes and mortality by voriconazole and amphotericin *The complete and partial responses were considered positive and the others were considered negative, including death within two and six weeks. IPA - invasive pulmonary aspergillosis (IPA)

Variable	Voriconazole (n=24)	Amphotericin (n=15)	p-value
2 weeks mortality	1 (4.17%)	0	-
6 weeks mortality	4 (16.67%)	1 (6.67%)	0.341
12 weeks mortality	6 (25%)	2 (13.33%)	0.178
IPA related mortality	0	0	-
Age (median)	45.8 (19.8)	42.0 (18.7)	0.077
Radiological Imagining			
Dense well-circumscribed lesions(s) with or without a halo sign	17 (70.83%)	14 (93.33%)	0.137
Air-crescent sign	1 (4.17%)	1 (6.67%)	0.22
Cavity	6 (25%)	0	0.038
Severity of disease and O_2_ requirement			0.158
Mild (not required oxygen therapy)	15 (62.50%)	7 (46.67%)	
Moderate (required oxygen therapy)	6 (25%)	6 (40%)	
Severe (required ventilation; non-invasive or mechanical ventilation)	3 (12.50%)	2 (13.33%)	
Global response*			0.124
Complete response	4 (16.67%)	1 (6.67%)	
Partial response	16 (66.67)	11 (73.33%)	
Disease worsening	1 (4.17%)	0	
Stable	0	1 (6.67%)	
Missing follow-up CT	3 (12.50%)	2 (13.33%)	
Stem cell transplant	11 (45.83%)	6 (40%)	0.137
Positive galactomannan	5 (20.83%)	3 (20%)	0.158
Neutropenia	8 (53.3%)	7 (29.2%)	0.326
Platelet count (median)	69.6 (117.3%)	85.1 (111.4%)	0.247
Side effects led to discontinuation	3 (12.50%)	1 (6.67%)	-

The dose of liposomal amphotericin B was 5 mg/kg in 66.67% of the patients, while the remaining patients were on 3, 3.75, 4, or 4.3 mg/kg. For voriconazole, 71.4% of the patients initially received an intravenous form of the medication, and the majority of the oral dose was 200 mg twice daily (90.91%), except for two patients who received 300 mg twice daily.

In total, nine side effects were reported for nine patients. For voriconazole, three patients reported hepatotoxicity, and one reported fever and skin rash. For liposomal amphotericin B, three patients reported electrolyte disruption, and two reported acute kidney injury. In five patients, the side effects led to the discontinuation of therapy (one had acute kidney injury, three had hepatotoxicity, and one had fever and skin rash). One patient was not included in the comparison analysis.

Follow-up CT was performed on a majority of the patients in both arms, except for three patients in the voriconazole arm and two patients in the liposomal amphotericin B arm. For voriconazole and liposomal amphotericin B, complete response was noticed in 16.67% and 6.67% of the patients, respectively, while the partial radiological response was noticed in 66.67% and 73.33% of the patients, respectively. Clinical response was aligned with radiological improvement, as all the patients who had complete or partial responses had clinical improvement.

Mortality and comparison between liposomal amphotericin B and voriconazole, and predictors of mortality

The six-week mortality was 6.67% (one out of 15) in the liposomal amphotericin B treatment arm compared to 16.67% (four out of 24) in the voriconazole arm. However, the 12-week mortality increased to 13.33% (two out of 15) and 25% (six out of 24) for these arms, respectively.

Successful responses, combining complete and partial responses, were not different between the two drugs: 80% (12 out of 15) for liposomal amphotericin B versus 83.33% (20 out of 24) for voriconazole.

Although some factors were associated with 12-week mortality (presence of dense, well‑circumscribed lesions(s) with or without a halo sign; HSCT recipient; absolute neutrophil count <0.500×109 cells/L; severity of disease), none of them were found to be significant in a multivariate analysis (see Table 3).

**Table 3:** Multivariate analysis of baseline factors of prognostic significance for mortality at 12 weeks

Variables	Odd ratio (95% CI)	p-value
Dense well-circumscribed lesions(s) with or without a halo sign in the radiological imaging	1.8 (0.1-25.1)	0.666
Success response versus negative	0.0 (0.0-5.8)	0.133
Stem cell transplant: yes versus no	0.0 (0.0-7.7)	0.192
Neutropenia: yes versus no	1.8 (0.1-34.1)	0.697
Severity of disease: severe versus other	2.8 (0.6-13.4)	0.2

IPA-related mortality was nil

The definition of IPA-related mortality was patients with stable disease or progression of disease at the time of death or patients with a partial response to treatment who died as the result of an event involving any of the sites of the original Aspergillus infection. Also, patients who died as a result of the toxicity of antifungal treatment [16].

The CT assessment of one patient indicated disease progression. This 40-year-old male had AML, failed stem cell transplant, and progressive pancytopenia with pericardial effusion, and he was declared eligible for supportive care and not for resuscitation. Four patients had no follow-up CT: an 82-year-old female patient with concomitant MDS/AML and TB, an 81-year-old male patient with AML and on palliative chemotherapy, a 65-year-old female patient with MDS and poor functional status, and a 54-year-old female patient with rheumatoid arthritis and diffuse large B-cell lymphoma who showed improvement after starting antifungal but later had a multiorgan failure and died.

## Discussion

In this single-center, retrospective cohort of IPA in hematological malignancy and stem cell transplant, we noticed eight out of 39 (20.51%) mortality at 12 weeks, but none of them was related to IPA. Within the same context, the severity of IPA was observed to be mild; surprisingly, more than half of the patients did not require oxygen therapy, and 43.47 % of the patients did not endorse respiratory symptoms throughout the course of IPA. 

IPA mortality in patients with hematological malignancy has decreased in the last 30 years, initially reported to be 60-80%, but in more recent data, it ranges between 15-30% [17,18].

While there is no large amount of data specifically about IPA-related mortality, a randomized controlled trial comparing combination therapy versus monotherapy for IPA reported a mortality of 19.3% for combination therapy and 27.5% for monotherapy; additionally, the IPA-related mortality was 88.5% and 84.6%, respectively, among overall mortality [16]. Another retrospective data comparing IPA in hematological malignancy versus solid organ transplant reported the six-week IPA‑attributable mortality in hematological malignancy to be 21% [19].

Within the same context, our reported global clinical response was higher than the previously reported one, which is between 40-61.9% [11,16,19].

Such discordance between the clinical response and IPA-related mortality when compared to previous studies, despite similar crude mortality, may be a result of a low number of patients in this study, which may have attenuated the significance of our finding, excluding the seven patients who were not eligible for comparing the two drugs thereby increasing the mortality to 26.08%. However, this may also reflect the continuous improvement in the care of IPA, justifying why the current real-life data likely has a lower fatality rate of IPA in those populations compared to previously reported data.

Furthermore, the milder severity of the disease noticed, which has not been reported in previous studies, indicates the disease is less fatal and thus has a better clinical response.

The current superiority of voriconazole to liposomal amphotericin B is thought to be questionable, as this superiority was established mainly in the trial performed by Hambrecht et al. [5]. That study was performed in 2002 and is thought to have flaws related to the following: the specific design in using the deoxycholate preparation form of amphotericin rather than the liposomal form, not limiting the Aspergillus site to the lung only, and the outcome measure likely not strongly supporting the current recommendation of using voriconazole as first-line therapy [20]. 

One can expect that liposomal amphotericin B is different than the conventional deoxycholate form since their pharmacokinetic/dynamic profiles were different within an in vitro lung model of IPA [21]. Evidently, in the serum, liposomal amphotericin B produces a higher peak concentration and area under the curve compared to deoxycholate [22].

While this current belief in the superiority of voriconazole has not been tested again in a head-to-head trial, the efficacy of the initial use of liposomal amphotericin B with different dosing was associated with a favorable response [23].

In our study, likely depending on IPA mortality in order to compare voriconazole to liposomal amphotericin B does not represent a fair comparison because the mortality was not directly associated with IPA. Additionally, the successful response was not different between the two drugs, although 33.33% of the patients were receiving less than the recommended dose of liposomal amphotericin B of 5 mg/kg.

Of note, there were more side effects in our cohort that led to discontinuation of voriconazole in three patients (10.71%) compared to one patient (6.67%) in the liposomal amphotericin B arm, despite that acute kidney injury with the new formulation of amphotericin is significantly less than with the prior conventional form [16,23].

The argument of low sensitivity is valid for diagnosing IPA in patients with hematological malignancy or HSCT recipients because the EORTC-MSG definition of invasive aspergillosis gives nonspecific radiologic findings an equal weight as that given by the aspergillus-specific mycologic criteria [18]. Thus, Nucci et al. suggested including patients with serologic test-supported invasive aspergillosis who were lacking the prespecified radiologic criteria, which are thought to have similar host, clinical, radiologic, and mycologic characteristics and outcomes of patients with compatible definitions [18]. Thus, a good percentage of patients with IPA would have nonspecific radiological findings, which was reported up to 47% [11]. In our inclusion of IPA cases, we adopted the old radiological criteria for diagnosis that do not include wedge-shaped and segmental or lobar consolidation, which is likely more specific for the IPA disease [24]. 

Preventive strategies for IPA have been tested; however, evidence suggested that there is no difference between preemptive strategy or universal prophylaxis with antifungal agents in terms of overall mortality or economic differences between the two approaches [25]. In our study, two patients failed voriconazole prophylaxis and developed IPA while on prophylaxis; additionally, one patient, who had a persistent fever for more than six days, reported failure of voriconazole as therapy and responded to liposomal amphotericin B within 48 hours after the start. Breakthrough fungal infection while using voriconazole has been mainly reported for candida and zygomycetes, except in one study that reported a 1/139 (1.43%) incidence of failure [26]. However, it was reported to be 17/305 (5.57%) in the clinical trial of voriconazole's comparison with fluconazole prophylaxis [27]. Nevertheless, it is difficult to define the exact effect of prophylaxis and breakthrough infection in our study because of the inconsistency of the prophylaxis strategy.

While the tight, ideal environment of clinical trials is difficult to achieve in a retrospective study (some miss follow-up radiology and switch from one medication to another), we overcame that by excluding this possible contamination by excluding the switching or patients on dual antifungal. Not to ignore the major limitation of the small number of patients. Low positivity of the galactomannan test in our cohort (19.5%) presents another limitation, which is major support for IPA diagnosis and reduced the number of included cases; however, we should consider that not all centers adopt galactomannan as the screening or confirmatory test. Reviewing by radiological diagnosis and judging on the follow-up by specialized radiologists are the strong points that give more validity and specificity to the findings compared to auto-extraction of the data. We believe that our result provides more insight into the real-life data of IPA, particularly when some centers are not applying the protocol (preemptive or universal prophylaxis). Thus, our data are more generalizable in signaling a favorable outcome of liposomal amphotericin, which needs to be tested or considered more in the future.

## Conclusions

IPA in hematological malignancy and stem cell transplant in this study's small cohort was noticed to have a milder presentation with no related mortality. In our cohort, the clinical and radiological IPA outcomes of using liposomal amphotericin B were not different from those of using voriconazole. However, a prospective study with a larger cohort or head-to-head trials is highly required to evaluate the current treatment regimens of IPA and assess their safety and efficacy.
